# Clinical and sociodemographic factors associated with time spent sitting in military police*

**DOI:** 10.1590/1980-220X-REEUSP-2023-0089en

**Published:** 2024-01-08

**Authors:** Cleise Cristine Ribeiro Borges Oliveira, Carla Tatiane Oliveira Silva, Ana Carla Carvalho Coelho, Bruna Rafaela Carneiro, Milena de Carvalho Bastos, Pollyanna Jorge Canuto, Mariana de Almeida Moraes, Fernanda Carneiro Mussi, Cláudia Geovana da Silva Pires

**Affiliations:** 1 Universidade Federal da Bahia, Programa de Pós-Graduação em Enfermagem e Saúde, Salvador, BA, Brazil.

**Keywords:** Police, Nursing, Sedentary Behavior, Motor Activity, Socioeconomic Factors, Policía, Enfermería, Conducta Sedentaria, Actividad Motora, Factores Socioeconómicos, Polícia, Enfermagem, Comportamento Sedentário, Atividade Motora, Fatores Socioeconômicos

## Abstract

**Objective:**

To verify the association between clinical and sociodemographic factors and time spent sitting in military police.

**Method:**

This is a cross-sectional study, with 432 military police officers from Eastern Regional Policing Command units of the Military Police of Bahia de Feira de Santana. Data collection took place from August to December 2022 through Google Forms using the International Physical Activity Questionnaire.

**Results:**

Men predominated (82.35%), race/color was black (87.04%), the head of the family had completed higher education (47.69%) and police officers with a partner (81.94%). The risk of time spent sitting ≥ 180 minutes per day was lower in males (IRR < 1). Increasing age was associated with a lower risk of time spent sitting ≥ 180 minutes per day (IRR < 1).

**Conclusion:**

Male police officers with more years of experience were less exposed to sedentary behavior. Specific interventions and health policies aimed at combating sedentary behavior become relevant, aiming to promote health and prevent diseases.

## RESUMO

**Objetivo:**

Verificar a associação entre fatores clínicos e sociodemográficos e o tempo gasto sentado em policiais militares.

**Método:**

Estudo transversal, com 432 policiais militares das unidades do Comando de Policiamento Regional Leste da Polícia Militar da Bahia de Feira de Santana. A coleta de dados ocorreu de agosto a dezembro de 2022 através do *Google Forms* constando o Questionário Internacional de Atividade Física.

**Resultados:**

Predominaram homens (82,35%), raça/cor negra (87,04%), nível de escolaridade do chefe da família superior completo (47,69%) e policiais com companheiro(a) (81,94%). O risco do tempo gasto sentado ≥ 180 minutos por dia foi menor no sexo masculino (IRR < 1). O aumento da idade foi associado a menor risco de tempo gasto sentado ≥ 180 minutos por dia (IRR < 1).

**Conclusão:**

Policiais do sexo masculino e com mais anos de vida estavam menos expostos ao comportamento sedentário. Intervenções específicas e políticas de saúde voltadas ao combate do comportamento sedentário se tornam relevantes, visando à promoção da saúde e prevenção de agravos.

## RESUMEN

**Objetivo:**

Verificar la asociación entre factores clínicos y sociodemográficos y el tiempo de permanencia en la policía militar.

**Método:**

Estudio transversal, con 432 policías militares de unidades del Comando de Policía Regional Este de la Policía Militar de Bahía de Feira de Santana. La recolección de datos se realizó de agosto a diciembre de 2022 a través de Google Forms utilizando el Cuestionario Internacional de Actividad Física.

**Resultados:**

Predominaron los hombres (82,35%), la raza/color fue negra (87,04%), el jefe de familia tenía estudios superiores (47,69%) y los policías con pareja (81,94%). El riesgo de pasar tiempo sentado ≥ 180 minutos por día fue menor en los hombres (IRR < 1). El aumento de la edad se asoció con un menor riesgo de pasar tiempo sentado ≥ 180 minutos por día (IRR < 1).

**Conclusión:**

Los policías varones con más años de experiencia estuvieron menos expuestos al comportamiento sedentario. Cobran relevancia intervenciones y políticas de salud específicas dirigidas a combatir el sedentarismo, con el objetivo de promover la salud y prevenir enfermedades.

## INTRODUCTION

Characteristics peculiar to the work activity of military police officers contribute to illness, especially cardiovascular diseases, which represent the leading cause of mortality in the world, being responsible for around 17.9 million deaths annually^(1)^. Modifiable cardiovascular risk factors (CVRF), such as smoking, excessive alcohol consumption, dyslipidemia, insufficient levels of physical activity, sedentary behavior and excess weight, are associated with the development of these diseases. Furthermore, daily contact with violence, crime, long working hours, fear of death and professional insecurity can generate stress and disorders related to mental health^(2,3)^.

Among CVRF, sedentary behavior estimated by time spent sitting, reclining or lying down is associated with all causes of mortality, regardless of regular physical activity, and deserves to be investigated regarding physical activity levels. Sedentary behavior can be characterized as any activity that reduces body energy expenditure to values close to resting levels, including activities such as sitting, sleeping, watching television and using the computer^(4)^.

Military police officers, even exercising a profession that requires good physical conditioning and regular physical activity, may exhibit sedentary behavior, given that the nature of work demands administrative activities and patrols carried out in a sitting position^(5)^. As the military police are one of the main bodies for ensuring the safety of society, police officers and their supervisors need to be aware not only of maintaining the troops’ physical fitness^(6)^, but also of the risks that sedentary behavior brings to health and the performance of work activities.

Regarding the state of the art, in the search in scientific literature in the Cochrane Central Register of Controlled Trials (CENTRAL), PubMed/National Library of Medicine, Virtual Health Library/BIREME and Education Resources Information Center (ERIC) databases, DeCS/MeSH descriptors were used (*Polícia*/Police OR Police Force; *Atividade Física*/Physical Activity; *Doença Cardiovascular*/Cardiovascular Disease; *Comportamento Sedentário*/Sedentary Behavior; *Educação em Saúde*/Health Education) in any language, with Boolean operators AND and OR. In the last ten years, no specific studies were found on sedentary behavior in military police officers. National and international research focused on physical activity and physical fitness in this professional category as well as the few educational programs aimed at analyzing these outcomes.

The gap in the literature makes it important to know how sedentary behavior is expressed in military police officers, aiming to direct interventions and public policies on healthy lifestyles for this group, which could contribute to improving health, personal and professional satisfaction^(7)^. It is noteworthy that sedentary behavior may be associated with clinical and sociodemographic factors, as found in other studies with other population groups^(8,9)^.

Based on the above, the present study aimed to verify the association between clinical and sociodemographic factors and time spent sitting in military police.

## METHOD

### Study Design and Place

This is a cross-sectional, analytical study, carried out from August 2022 to December 2022, in all organic Eastern Regional Policing Command (Eastern CPR) organic units of the Military Police of Bahia (PMBA), based in the city of Feira de Santana, namely: Eastern CPR; School Policing; Ronda Maria da Penha; Independent Tactical Police Company (Rondesp/East); 64^th^ Independent Police Company (CIPM); 65^th^ CIPM; 66^th^ CIPM; and 67^th^ CIPM.

### Study Sample and Sample Calculation

The study sample consisted of 432 military police officers from all Eastern CPR units of Feira de Santana, including the category of enlisted personnel (soldier, corporal, sergeant and warrant officer) and officers (midshipman, lieutenant, captain, major, lieutenant colonel and colonel). All categories had a minimum working hours of 40 hours per week. During daily workday, everyone is instructed to perform some type of physical activity, but there is no supervision regarding compliance. Periodically, all of the corporation’s professionals, from both categories, undergo a physical fitness test.

The sample calculation was carried out considering a 5% sampling error (α = 0.05), 95% confidence interval (1 – β = 0.95) and a prevalence of sedentary lifestyle of 37.25%^(10)^ according to previously prepared studies. This sample N was adopted when considering that it was not a question of collection in a single location, that is, a conglomerate study design, in which police officers from different CPRs participated in the research. The formula n = N.Z2.p.(1 – p) / Z2.p.(1 – p) + e^2^.N – 1 was adopted, where: na: calculated sample; The population; E: normal variable; p: real probability of an event; e: sampling error. Thus, a sample of 428 participants was estimated.

### Instruments and Data Collection

Data collection was carried out using a form built in Google Forms containing the questions from the International Physical Activity Questionnaire (IPAQ). The IPAQ is a validated instrument, developed by the World Health Organization through the Centers for Disease Control and Prevention and aims to estimate the usual level of physical activity and sedentary behavior in populations from different countries and sociocultural contexts, and has been validated for use in Brazil^(11)^. In this questionnaire, in section number five, there are questions related to sedentary behavior (measurements of sitting time) that were answered by police officers: “How much time in total do you spend sitting during a weekday?” and “How much time in total do you spend sitting during a weekend day?” Police officers should remember in a typical day the time in hours spent sitting at work, at home and during free time, such as resting, reading or watching television, not taking into account time spent sitting on the bus, train, subway or car. Hours were transformed into minutes.

Time spent sitting was calculated as follows: time spent sitting during a weekday (Monday to Friday) in minutes **×** 5 added to time spent sitting during the weekend **×** 2, divided by seven. Time spent sitting elevated was considered for police officers who sat ≥180 minutes/day^(12)^.

Additionally, the instrument was composed of clinical variables (diagnosis of hypertension, dyslipidemia and coronary artery disease (CAD)) and sociodemographic variables (age, sex, self-declared color, education, marital status, income, number of people who depend on income and monthly expense).

Participants were invited to participate in the research after authorization from the Military Police Command. After acquiescence, Google Forms was sent to participants’ WhatsApp. One of the researchers attended police units during the week to explain the objective of the investigation and clarify possible doubts regarding filling out the information.

### Statistical Analysis

All collected variables were subjected to descriptive analyses. For categorical variables, absolute (n) and relative (%) frequencies were calculated. For numerical variables, the mean, median, standard deviation, quartiles 1 and 3 (which correspond, respectively, to the 25^th^ and 75^th^ percentiles) and the minimum and maximum values were calculated.

To assess the association between time spent sitting and sociodemographic and clinical variables, hypothesis tests were carried out. For nominal categorical sociodemographic and clinical variables, the chi-square test of independence was used, since the data met the assumptions of this test (expected frequencies greater than 5 in at least 80% of cells and 100% of cells with expected frequencies greater than 5 to 1)^(13)^. Statistically significant chi-square or Fisher’s exact tests were followed by analysis of adjusted standardized residuals (Pearson’s r residuals) to identify in which categories the observed frequencies differed from those expected. Residuals outside the range [–1.96; 1.96] were considered statistically significant^(14)^. For sociodemographic and numeric or ordinal clinical variables, the Mann-Whitney test was used.

Due to the impact of sample size on p-value^(15)^, effect size measures were calculated for all tests. For the Mann-Whitney test, the effect size r was calculated, which can be classified as small (r > 0.1), medium (r > 0.3) or large (r > 0.5)^(16)^.

For the chi-square test of independence, Cramer’s V effect size was calculated, whose classification depends on the degrees of freedom^(16)^. The degrees of freedom for Cramer’s V correspond to the minimum value between the number of rows and the number of columns of the cross-reference table minus one. The classification is described in Table 1.

**Table 1:** Classification suggested by Cohen^(16)^ for Cramer’s V effect size according to degrees of freedom (df) – Salvador, BA, Brazil, 2022.

df	Derisory	Small	Medium	Large
1	<0.10	<0.30	<0.50	≥0.50
2	<0.07	<0.21	<0.35	≥0.35
3	<0.06	<0.17	<0.29	≥0.29
4	<0.05	<0.15	<0.25	≥0.25
5	<0.04	<0.13	<0.22	≥0.22

For Cramer’s V, the degrees of freedom (df) depend on the size of the cross-reference table, corresponding to the minimum value between: number of rows – 1 and number of columns – 1.

In the multivariate way to assess Incidence Risk Ratio (IRR) associated with time spent sitting, the Poisson regression model was applied, with robust standard errors (obtained by the Huber-White estimator)^(17)^. This model included time spent sitting as a dependent variable and age, sex, diagnosis of hypertension, diagnosis of CAD and diagnosis of dyslipidemia as independent variables.

Poisson regression coefficients with robust standard errors, when exponentiated, result in relative risks. IRRs that do not statistically differ from 1 (which, therefore, include the value 1 in their 95% confidence interval) indicate that that particular independent variable has no impact on the risk of the outcome occurring – in this case, time spent sitting ≥180 minutes per day. IRRs statistically greater than 1 indicate an increased risk of the outcome occurring, while IRRs statistically less than 1 indicate a reduction in this risk.

For numerical independent variables, IRR indicates the expected change in risk for each unit increase in the independent variable. For categorical independent variables, the interpretation of IRR needs to take into account the reference category: IRR indicates the observed change in the risk of occurrence of the outcome when participants belong to that particular category versus when they belong to the reference category.

All analyzes were conducted using R software version 4.1.0^(18)^, and considered a significance level of 5%.

### Ethical Aspects

The research followed the specifications of Resolution 466/12 and Resolution 510/16 of the Brazilian National Health Council, which regulate research involving human beings^(19,20)^. It follows the guidelines for research in a virtual environment from the Brazilian National Research Ethics Council, in accordance with Circular Letter 2/2021/CONEP/SECNS/MSA, which provides guidelines for procedures in research with any stage in a virtual environment. It was submitted to the Research Ethics Committee, and was approved in August 2022, under Opinion 5,577,350.

All those invited to the research were previously informed about the objectives, justifications as well as the risks and benefits involved with participation. Consent was obtained from each guest who agreed to participate in the research, through the Informed Consent Form (ICF), which was also inserted in Google Forms, preceding the questions.

## RESULTS

The sample consisted of 432 participants. The predominance was male (82.35%), black race/color (black and brown) (87.04%), level of education of the head of the family higher education (47.69%) and with a partner (81, 94%). The means verified were 39.31 years of age, 6.09 minimum wages of monthly income, 3.28 people who depended on the monthly income and R$ 4,596.41 (US$919.28) of monthly expenses.

Regarding personal history of cardiovascular risk, there was a predominance of people without hypertension (83.10%), dyslipidemia (49.07%) and without CAD (86.81%).

It was observed that the age values of the group with time spent sitting ≥180 minutes per day tended to be lower than the group whose time spent sitting was less than 180 minutes per day (W = 1.5676.5; p = 0.020; r = 0.112). The observed effect size r can be classified as small. These results are detailed in Table 2.

**Table 2:** Factors associated with time spent sitting in military police Eastern Regional Policing Command organic units in Feira de Santana from August to December 2022 – Salvador, BA, Brazil, 2022 (N = 432).

Variable	Time sitting <180 min/day (n = 75)	Time sitting ≥180 min/day (n = 357)	p	TE
**Sex**			0.190^1^	0.063
Female	8 (10.67)	63 (17.65)		
Male	67 (89.33)	294 (82.35)		
**Age**			0.020^2^	0.112
Mean (SD)	41.12 (7.30)	38.93 (7.34)		
Median (Q1 – Q3)	42.00 (36.00–46.50)	39.00 (34.00–44.00)		
**Race/color**			0.769^1^	0.014
Non-black	11 (14.67)	45 (12.61)		
Black (balck and brown)	64 (85.33)	312 (87.39)		
**Education of the head of the family**			0.613^2^	0.024
Median (Q1 – Q3)	4.00 (4.00–5.00)	4.00 (4.00–5.00)		
No formal education/Incomplete elementary school I	4 (5.33)	16 (4.48)		
Complete elementary school I/ Incomplete elementary school II	1 (1.33)	15 (4.20)		
Complete elementary school II/ Incomplete high school	1 (1.33)	10 (2.80)		
Complete high school/Incomplete higher education	32 (42.67)	147 (41.18)		
Complete higher education	37 (49.33)	169 (47.34)		
**Family monthly income**			0.119^2^	–0.075
Mean (SD)	6.03 (6.39)	6.11 (4.40)		
Median (Q1 – Q3)	5.00 (3.00–6.00)	5.00 (4.00–7.00)		
**Number of people who depend on income**			0.081^2^	0.084
Mean (SD)	3.52 (1.46)	3.23 (1.24)		
Median (Q1 – Q3)	4.00 (2.00–4.00)	3.00 (2.00–4.00)		
**Monthly income**			0.165^2^	–0.067
Mean (SD)	4410.93 (3112.34)	4635.38 (2535.14)		
Median (Q1 – Q3)	4000.00 (3000.00–5000.00)	4000.00 (3000.00–5000.00)		
**Marital status**			1.000^1^	0.000
With partner	61 (81.33)	293 (82.07)		
Without partner	14 (18.67)	64 (17.93)		
**Hypertension**			0.338^1^	0.046
No	59 (78.67)	300 (84.03)		
Yes	16 (21.33)	57 (15.97)		
**Coronary artery disease**			0.913^1^	0.021
No	66 (88.00)	309 (86.55)		
Do not know	4 (5.33)	19 (5.32)		
Yes	5 (6.67)	29 (8.12)		
**Dyslipidemia**			0.549^1^	0.053
No	31 (41.33)	172 (48.18)		
Do not know	3 (4.00)	14 (3.92)		
Yes	41 (54.67)	171 (47.90)		

1. Chi-square test of independence; 2. Mann-Whitney test; ES = effect size. The following effect sizes were calculated: r, for the Mann-Whitney test; Cramer’s V, for the chi-square test of independence.

There was no statistically significant association between time spent sitting and other sociodemographic and clinical variables. These results are detailed in Table 2.

Poisson regression indicated that age and sex are factors statistically associated with time spent sitting. The results indicated that the risk of belonging to the group of time spent sitting ≥180 minutes per day is lower among males (IRR < 1) when compared to females (reference category). With respect to age, increasing age was associated with a lower risk of time spent sitting ≥180 minutes per day (IRR < 1). These results are detailed in Table 3.

**Table 3:** Poisson regression with robust estimator with time spent sitting as dependent variable in military police officers from Eastern Regional Policing Command organic units from August to December 2022 in Feira de Santana – Salvador, BA, Brazil, 2022 (N = 432).

Independent variable	IRR	95% CI	P
**Dyslipidemia**			
No	–	–	
Do not know	1.000	0.902; 1.110	0.995
Yes	0.992	0.952; 1.035	0.721
**Coronary artery disease**			
No	–	–	
Do not know	1.003	0.922; 1.090	0.951
Yes	1.034	0.964; 1.109	0.347
**Hypertension**			
No	–	–	
Yes	0.996	0.936; 1.059	0.887
**Sex**			
Female	–	–	
Male	0.950	0.903; 0.999	0.045
**Age**	0.996	0.993; 1.000	0.025

IRR = Incidence Risk Ratio; CI = confidence interval.

## DISCUSSION

For the vast majority of military police officers, time spent sitting greater than or equal to 180 minutes per day (82.6%) was identified, showing exposure to this cardiovascular risk factor in a group predominantly in the young age group. In the work environment, remaining in a sitting position occurs both in the car, carrying out patrols and in administrative activities^(8)^. During the workday, they spend most of their time sitting, with infrequent peaks of intense activities, carrying weight on their bodies due to their uniforms, protective equipment, such as bulletproof vests and weapons used^(21)^, contributing to sedentary behavior, which can trigger cardiometabolic diseases^(22)^.

Considering that around 49.07% had dyslipidemia, a possible aggregation of CVRF was observed in the sample studied, which reveals the need for prevention and control measures. The reduced lipoprotein lipase activity observed in waking behaviors that require low energy expenditure, in the range of 1.0 to 1.5, metabolic equivalent/s (MET), in a sitting, reclining or lying position, with the exception of sleeping hours, is associated with increased triglyceridemia, low HDL levels, hypertension, metabolic syndrome, among others^(23)^. Furthermore, sedentary behavior is associated with the development of several diseases and premature mortality, increasing the risk of dying by 50 times^(5)^.

The military police officer’s workplace needs to be considered a space for the development of healthy habits, including less sedentary activities, regardless of the role performed, given that the daily time dedicated to work activities is at least 40 hours per day. Although police officers are encouraged to carry out physical activity within their working hours, they also need to be the target of actions aimed at combating excessive time spent sitting, lying down or reclining. They should also be encouraged not to perform daily activities that do not increase energy expenditure substantially above the resting level, such as using the computer, working in a sitting position, among other screen-based behaviors.

In the sample of this present study, the police officers most exposed to time spent sitting greater than or equal to 180 minutes per day were female and younger. In this regard, the planning of strategic programs to encourage mobility, such as standing up every hour they spend sitting, can protect them from the negative impacts of sedentary behavior, being a priority and needing to gain visibility within military corporations as a whole. For instance, an intervention study, with 24 police officers who worked in a police office, assessed the theory derived from the sedentary intervention in the workplace, observing improvements in sitting and standing in the workplace, weight loss and team relationships. Furthermore, participants considered the interventions highly acceptable and practicable in everyday life as protective measures against cardiovascular diseases^(24)^.

The data from this study showed the scarcity of operational service of female military personnel who are increasingly directed only to administrative activities in the military service, favoring sedentary behavior. The smaller number of women in the study may also reflect the difference in the availability of vacancies between sexes for police competitions, reinforcing the inequalities inherent to sexual division of labor both in restricted occupations of women and in disadvantage in the type of work performed, in wages, in professional career and in working conditions^(20)^.

Physical activity is intrinsic to military police officers’ profession, as, as public security agents, they must be capable of pursuit, whether with vehicles, motorcycles, on horseback, or on foot^(5)^. Thus, the attention of military corporations is focused on physical conditioning, in particular strength and cardiorespiratory fitness, to meet work demands. These corporations need to encourage physical activity not only to indoctrinate their bodies, but to preserve life, also incorporating as a goal the incentive to combat sedentary behavior during workday.

The military corporation’s involvement in encouraging police officers to lead a healthy life is essential for workers’ health and the efficient performance of their activities and, consequently, for the quality of services offered by the institution^(3)^. A solid intervention to raise awareness among the corporation, politicians and health managers is necessary, aiming at the implementation of specific programs related to encouraging adherence to healthy lifestyles by military police officers^(2)^, especially aimed at the most vulnerable groups, such as women and younger people, giving visibility to police health promotion^(8)^.

The results of this study must be interpreted with caution, as the sample reflects the local characteristics of the police officers studied, limiting data extrapolation. Furthermore, although the instrument used to collect data is validated in the country, it was self-completed by police officers via a digital platform, which may underestimate or overestimate time spent sitting. A set of occupational variables was not explored in the study, and deserves attention in other investigations, as it may influence the outcome investigated. The originality of this study is highlighted, as it is the first to investigate sedentary behavior in military police officers. For this reason, it was difficult to compare the results with other studies.

## CONCLUSION

A high percentage of military police officers were exposed to time spent sitting for more than or equal to 180 minutes per day, especially those who were female and younger. Specific interventions to reduce time spent sitting during work activities are essential. These actions could support future public policies on healthy lifestyles for military police officers, enabling improvements in health indicators and prevention of injuries.
